# Global healthcare use by immigrants in Spain according to morbidity burden, area of origin, and length of stay

**DOI:** 10.1186/s12889-016-3127-5

**Published:** 2016-05-27

**Authors:** Luis A. Gimeno-Feliu, Amaia Calderón-Larrañaga, Esperanza Diaz, Beatriz Poblador-Plou, Rosa Macipe-Costa, Alexandra Prados-Torres

**Affiliations:** EpiChron Research Group on Chronic Diseases, Aragón Health Sciences Institute (IACS), IIS Aragón, Miguel Servet University Hospital, Zaragoza, Spain; San Pablo Health Centre, C/ Aguadores 7, 50003 Zaragoza, Spain; Department of Medicine, Psychiatry and Dermatology, University of Zaragoza, Zaragoza, Spain; Red de Investigación en Servicios de Salud en Enfermedades Crónicas (REDISSEC), Carlos III Health Institute, Madrid, Spain; Department of Public Health and Primary Health Care, University of Bergen, Bergen, Norway; Norwegian Centre for Minority Health Research (NAKMI), Oslo University Hospital, Bergen, Norway; Department of Microbiology, Preventive Medicine and Public Health, University of Zaragoza, Zaragoza, Spain

## Abstract

**Background:**

The healthcare of immigrants is an important aspect of equity of care provision. Understanding how immigrants use the healthcare services based on their needs is crucial to establish effective health policy.

**Methods:**

This retrospective, observational study included the total population of Aragon, Spain (1,251,540 individuals, of whom 11.9 % were immigrants). Patient-level data on the use of primary, specialised, hospital, and emergency care as well as prescription drug use in 2011 were extracted from the EpiChron Cohort and compared between immigrants and nationals. Multivariable standard or zero-inflated negative binomial regression models were generated, adjusting for age, sex, length of stay, and morbidity burden.

**Results:**

The annual visit rates of immigrants were lower than those of nationals for primary care (3.3 vs 6.4), specialised care (1.3 vs 2.7), planned hospital admissions/100 individuals (1.6 vs 3.8), unplanned hospital admissions/100 individuals (2.7 vs 4.7), and emergency room visits/10 individuals (2.3 vs 2.8). Annual prescription drug costs were also lower for immigrants (€47 vs €318). These differences were only partially attenuated after adjusting for age, sex and morbidity burden.

**Conclusion:**

In a universal coverage health system offering broad legal access to immigrants, the global use of healthcare services was lower for immigrants than for nationals. These differences may be explained in part by the healthy migration effect, but also reveal possible inequalities in healthcare provision that warrant further investigation.

**Electronic supplementary material:**

The online version of this article (doi:10.1186/s12889-016-3127-5) contains supplementary material, which is available to authorized users.

## Background

While immigration is an ancient social phenomenon, its influence on health policy in developed countries has increased in recent decades [1]. In 2010, an estimated 72.6 million migrants were living in the WHO European region [2]. According to the WHO, governments must ensure that migrants are entitled to health services, that services are appropriate to their needs, and that information systems are in place to monitor utilisation and detect inequities [3, 4]. Indeed, in recent years developed countries have made significant efforts to measure the actual impact of immigration on healthcare systems [3, 5–8]. However, while the WHO’s statements recommend improvements to universal coverage [3, 9], some of the world’s richest countries have moved in the opposite direction, limiting access to healthcare [10], especially for immigrants [2, 4, 11, 12].

Knowledge about immigrants’ use of health systems has been inconsistent. While most studies point to lower utilisation rates among immigrants [6, 13–17], others have shown the reverse [18]. A review by Norredam and coworkers found higher utilisation rates in immigrants as compared with nationals [7], but noted a lack of appropriate epidemiological data and inconsistencies in the methods used to categorise immigrants across studies. These differences may be due to socio-economic disparities across immigrant groups in different countries [19], varying health levels or health cultures [20, 21], and/or differing barriers to access at patient, provider and system levels [22, 23]. However, one of the main pitfalls of the existing literature is the inclusion of only one level of healthcare at a time, for example use of emergency room services [24], which prevents us from acquiring a comprehensive picture of the use of the healthcare system.

Morbidity is a useful factor to detect possible imbalances between availability, needs, and utilisation rates [6, 17, 20]. Population-based studies that include objective and comprehensive data on clinical encounters, diagnoses, and prescriptions at the individual level allow us to adjust use rates by health needs, and to avoid selection and/or response biases [25].

This study analyses the global use of healthcare services (i.e., primary care, specialised care, hospitals, emergency room, and prescription drug use) by immigrants to Spain as compared with Spanish nationals, considering morbidity level and demographic characteristics of the immigrants.

## Methods

This cross-sectional population-based retrospective study included individuals assigned to all public primary care (PC) centres in Aragon, Spain, during 2010 and 2011. The Aragon Health Service is part of the Spanish National Health System, which offers universal coverage and is almost fully funded by taxes. Care provision is free of charge at the point of delivery, resulting in a practically free system [26]. PC centres serve as gatekeepers and are distributed so as to guarantee appropriate geographical coverage. Secondary care is provided through ambulatory specialised care, hospitals, and emergency rooms. Pharmaceuticals prescribed to those under 65 require a co-payment of 40 % of the retail price (or less in the case of chronic medication); medications are otherwise free of charge at the point of delivery. At the time our study was conducted, immigrants were guaranteed legal access to the same healthcare services as Spanish nationals, regardless of their legal status [26]. Immigrants in Spain account for 12.2 % of the population (12.7 % in Aragon) and migrate to Spain primarily for economic reasons [27].

The data used in this study correspond to year 2011. Socio-demographic variables (i.e., age, sex, nationality, rurality and length of stay in Spain) were extracted from patients’ health insurance cards. Immigrants were defined as persons of non-Spanish nationality, regardless of their place of birth and length of stay in Spain. Six areas of origin were distinguished based on the nationalities of the study population: Spain, Asia, Africa, Eastern Europe, Latin America, and Western Europe/North America.

Diagnostic data was extracted from PC electronic health records and from the Hospital Minimum Basic Dataset (Spanish acronym, CMBD). In the former, diseases are registered according to the International Classification of Primary Care, Version 1 (ICPC-1). The latter registers the diagnoses of patients discharged from all public and private hospitals in Aragon, coded using the Clinical Modification of the Ninth Revision of the International Classification of Diseases (ICD-9-CM). The ACG System® was used to group all ICPC and ICD diagnostic codes based on their duration, severity, diagnostic certainty, aetiology, and specialty care involvement. A unique ACG (Adjusted Clinical Group) category was assigned to each individual based on age, sex, and all diagnoses registered during the study period. Individuals within a given ACG show similar patterns of morbidity and resource utilisation over a given year. For the sake of parsimony, ACGs with a similar expected use of resources were aggregated into one of the six so-called Resource Utilisation Bands (RUB 0 = non users; RUB 1 = healthy users; RUB 2 = low morbidity; RUB 3 = moderate morbidity; RUB 4 = high morbidity; and RUB 5 = very high morbidity). Each individual was thus additionally assigned a RUB category.

The use of PC was measured as the number of visits to the PC doctor and nurse including those on demand, scheduled, emergency, and home visits. Specialised care utilisation was measured as the total number of visits to any specialist. Hospital care use included planned and unplanned admissions, and the total number of hospital days. The use of emergency room services was measured as the total number of visits and priority visits. Priority visits were identified based on the triage level established by the Aragon Health Service; out of the five categories listed, levels 1–3 are assigned to priority visits. Prescription drug use was measured as the total annual expense using recommended retail drug prices [15].

Data used for this study are part of the EpiChron Cohort, which gathers demographic, clinical, and pharmaceutical information of patients living in Aragon, matched through a unique anonymized personal identification code. The study was approved by the Clinical Research Ethics Committee of Aragon (CEICA).

### Statistical analysis

The mean number of visits to each level of care and average prescription drug use was calculated by area of origin. Given the over-dispersion in the distribution of the outcome variables, negative binomial regression models were applied to determine the association between the latter and area of origin. To verify the adequacy of the negative binomial regression models with respect to the normal Poisson count models, the over-dispersion parameter alpha was assessed. Because the observed outcome data often displayed a higher relative frequency of zeros than is consistent with the negative binomial model specifications, zero-inflated models were used. Vuong test statistics were checked to provide the appropriateness of zero-inflated models against the standard negative binomial models. When the Vuong test was statistically non-significant showing large negative values, standard negative binomial models were employed. The test statistics are available upon request to the authors. When non-concave regions repeatedly appeared, an alternative stepping algorithm to the standard maximum likelihood algorithm was permitted.

Different models were built for each of the six outcome variables (i.e., visits to the PC doctor, visits to specialised care, total admissions, unplanned admissions, visits to the emergency room, and prescription drug costs) area of origin being the main independent variable. All models were first adjusted for age and sex, and then for age, sex, and morbidity burden (RUB categories), and were stratified by age group (i.e., ≤ 14 and > 14 years) and by immigrants’ length of stay in Spain (i.e., < 5 and ≥ 5 years). The 5-year time cut-off point was chosen based on previous literature [28–30].

Differences in the use of emergency care and hospital care (unplanned admissions) were also analysed after stratifying the regression models by PC users and non-users. Furthermore, urban/rural differences in the use of healthcare services were measured by including this independent variable in the standard or zero-inflated negative binomial regression models, depending on the output of the Vuong test. Incidence rate ratios (IRR) and their 95 % confidence intervals were graphically represented.

All statistical analyses were conducted using STATA/IC 12.

## Results

We analysed data from 1,251,540 persons, 11.9 % of whom were immigrants. Of these, 148,756 were children (14.3 % immigrants). Immigrant distribution according to country of origin is shown in Additional file 1.

Demographic characteristics, morbidity burden and rates of use of healthcare services are presented in Table 1. Immigrants were younger and had a lower morbidity burden, and a greater proportion of immigrants were categorised as healthy users or non-users as compared with nationals. A lower percentage of immigrants visited PC centres during the study period (average visits to doctor and nurse: 3.3 and 0.8 times per year, respectively, for immigrants; 6.4 and 3.3 per year for nationals). In the case of urgent visits to PC, rates were higher for Africans compared with nationals. The use of specialised ambulatory appointments, the number of hospitalisations (planned and unplanned) and the mean hospital stay were lower for immigrants. A lower percentage of immigrants visited the emergency room and they made fewer visits on average than nationals. Emergency visits by immigrants were more frequently classified as low priority by the triage system. The mean prescription drug cost per patient was 6.8 times higher for nationals than for immigrants.

**Table 1:** Demographics, morbidity burden, and healthcare service use for nationals and immigrants in Spain

	Nationals	Immigrants	Latin America	Eastern Europe	Africa	Asia	Western Europe & North America
N	1,102,391	149,149	41,060	56,011	37,603	5,723	8,752
Demographic information							
0-14 years, %	11.6	14.2	11.0	13.6	18.8	18.2	11.1
15-44 years, %	36.8	67.3	68.2	68.7	67.4	63.7	55.5
45-64 years, %	27.7	17.0	18.7	17.0	12.9	15.9	27.5
65 years +, %	23.9	1.5	2.1	0.7	0.9	2.2	5.9
Women, %	51.1	46.1	56.4	48.2	33.4	44.7	40.6
Rural, %	39.8	37.4	23.6	46.8	38.8	18.1	49.2
Length of stay in Spain ≥5 years, %	---	59.0	60.0	58.4	59.8	49.2	61.0
Morbidity burden							
Healthy users/non-users, %	22.1	43.0	37.5	46.7	40.5	52.9	50.7
Low/moderate morbidity, %	71.7	54.4	59.8	51.1	56.6	45.1	46.8
High/very high morbidity, %	6.2	2.6	2.7	2.3	3.0	2.0	2.5
Use of Primary Care							
No visits to doctor, %	22.2	37.8	34.9	41.1	31.5	46.5	52.1
No visits to nurse, %	48.7	73.0	72.0	75.4	69.0	79.5	75.8
Mean (SD) No. of visits to doctor, normal care	6.4 (8.1)	3.3 (5.0)	3.7 (5.1)	3.0 (4.9)	3.7 (5.1)	2.5 (4.3)	2.7 (5.1)
Mean (SD) No. of visits to doctor, urgent care	0.4 (1.1)	0.4 (1.1)	0.3 (0.9)	0.4 (1.1)	0.6 (1.4)	0.3 (1.0)	0.2 (0.9)
Mean (SD) No. of visits to nurse	3.3 (7.2)	0.8 (2.9)	0.8 (2.7)	0.7 (2.8)	0.9 (2.8)	0.7 (3.7)	1.0 (3.3)
Use of Specialised Care							
No visits, %	48.3	75.6	59.3	70.6	71.1	74.1	75.7
Mean (SD) No. of visits	2.7 (4.7)	1.3 (3.2)	1.7 (3.5)	1.2 (3.0)	1.2 (3.0)	1.0 (2.8)	1.1 (3.2)
Use of Hospital Care							
Mean (SD) No. of planned admissions/100 ind.	3.8 (25.1)	1.6 (30.0)	2.0 (20.7)	1.5 (17.5)	1.4 (50.8)	0.8 (10.0)	1.7 (14.6)
Mean (SD) No. of unplanned admissions/100 ind.	4.7 (27.0)	2.7 (17.7)	2.8 (18.8)	2.5 (17.9)	3.2 (19.9)	2.8 (18.7)	2.2 (17.9)
Mean (SD) hospital stay, days	6.5 (8.3)	4.3 (9.3)	4.3 (5.9)	4.4 (13.3)	4.0 (6.0)	4.5 (5.7)	5.5 (9.2)
Use of Emergency Care							
No visits, %	82.2	85.2	82.5	85.7	85.9	85.8	90.6
Mean (SD) No. of visits/10 ind.	2.8 (7.8)	2.3 (7.2)	2.7 (7.4)	2.3 (7.0)	2.3 (7.4)	2.3 (7.2)	1.4 (5.5)
High priority visits, %	48.9	37.2	40.9	36.5	33.6	31.3	42.2
Pharmacy use							
No use, %	24.8	46.2	43.7	49.9	39.3	53.9	58.9
Mean (SD) cost, €	317.5 (716.2)	47.0 (220.0)	50.9 (201.7)	42.3 (200.4)	43.5 (230.3)	33.4 (139.6)	82.0 (367.0)

*SD* Standard deviation, *Ind*. Individuals

Table 2 shows the incidence rate ratios for the use of healthcare services by immigrants with respect to nationals, first adjusting for age and sex, and then for age, sex, and morbidity burden. The data are presented for children and adults separately, and disaggregated by area of origin and length of stay. Except for African adults living for long periods in Spain, immigrants showed lower PC use than nationals, even after adjusting for morbidity burden, although this adjustment diminished the differences observed for all immigrant groups. Immigrants from Asia and Western Europe/North America showed the lowest rates of PC use. For both specialised ambulatory care and prescription drug costs, use rates were significantly lower for immigrants, regardless of area of origin, age group, and length of stay. During their first five first years in the region, immigrant children, particularly those from Africa, were hospitalised significantly more often than Spanish children. After five years of residence in the region, the admission rate for African children was significantly lower and that of Latin American children was significantly higher than that of Spanish children. Adult immigrants were hospitalised less often than nationals regardless of their length of stay in the region. Finally, the use of emergency room services was generally lower for immigrant children, although differences were observed across areas of origin. Asian children used emergency room services more often than Spanish children, while African children used them less often. Immigrant children visited the emergency department less often than their national counterparts, even when their length of stay in the region was over five years. Among immigrant adults, the use of emergency room services was higher than that of nationals, with Latin Americans showing the highest relative rates. Finally, immigrants’ prescription drug use was lower than that of nationals regardless of age, nationality, or length of stay.

**Table 2:** Use of healthcare services (incidence rate ratios, IRR). Results of standard or zero-inflated negative binomial regression models^¶^ across immigrant groups

	Use of Primary Care (visits to doctor)		Use of Specialised Care		Use of Hospital Care (total admissions)		Use of Hospital Care (unplanned admissions)		Use of Emergency Care		Pharmacy Use	
	IRR^a^	IRR^b^	IRR^a^	IRR^b^	IRR^a^	IRR^b^	IRR^a^	IRR^b^	IRR^a^	IRR^b^	IRR^a^	IRR^b^
Length of stay in Spain < 5 years												
Children												
Nationals (ref)	1.00											
Immigrants	0.79^†^	0.93^†^	0.59^†^	0.70^†^	1.02	1.30^†^	0.83*	1.03	0.76^†^	0.92*	0.66^†^	0.71^†^
Africa	0.80^†^	0.95^†^	0.57^†^	0.67^†^	1.45^†^	1.81^†^	0.95	1.15	0.59^†^	0.69^†^	0.75^†^	0.87^†^
Asia	0.59^†^	0.83^†^	0.39^†^	0.55^†^	0.32*	0.51	0.43*	0.69	1.03	1.50^†^	0.35^†^	0.41^†^
Eastern Europe	0.84^†^	0.96^†^	0.55^†^	0.65^†^	0.81*	1.01	0.81	0.99	0.89*	1.07	0.66^†^	0.70^†^
Latin America	0.78^†^	0.92^†^	0.75^†^	0.88*	1.01	1.24	0.77	0.96	0.78^†^	0.92	0.65^†^	0.64^†^
Western Europe & North America	0.52^†^	0.69^†^	0.45^†^	0.57^†^	0.54	0.8	0.68	1.0	0.60^†^	0.79	0.41^†^	0.40^†^
Adults												
Nationals (ref)	1.00											
Immigrants	0.68^†^	0.88^†^	0.60^†^	0.71^†^	0.78^†^	0.91^†^	0.95	1.03	0.87^†^	1.05*	0.37^†^	0.41^†^
Africa	0.88^†^	1.06^†^	0.68^†^	0.72^†^	1.02	1.05	1.42^†^	1.36^†^	0.96	1.07*	0.40^†^	0.45^†^
Asia	0.46^†^	0.75^†^	0.52^†^	0.65^†^	0.74*	0.88	1.1	1.2	0.82*	1.12	0.26^†^	0.33^†^
Eastern Europe	0.57^†^	0.80^†^	0.52^†^	0.65^†^	0.67^†^	0.83^†^	0.79^†^	0.89*	0.77^†^	0.98	0.34^†^	0.37^†^
Latin America	0.76^†^	0.88^†^	0.84^†^	0.99	0.84^†^	0.96	0.88*	0.97	1.02	1.19^†^	0.40^†^	0.45^†^
Western Europe & North America	0.44^†^	0.69^†^	0.39^†^	0.49^†^	0.40^†^	0.54^†^	0.47^†^	0.58*	0.47^†^	0.62^†^	0.38^†^	0.36^†^
Length of stay in Spain ≥ 5 years												
Children												
Nationals (ref)	1.00											
Immigrants	0.77^†^	0.90^†^	0.61^†^	0.69^†^	0.79*	0.93	0.71*	0.84	0.69^†^	0.79^†^	0.55^†^	0.60^†^
Africa	0.80^†^	0.90^†^	0.56^†^	0.62^†^	0.53^†^	0.58^†^	0.62*	0.7	0.54^†^	0.60^†^	0.58^†^	0.58^†^
Asia	0.63^†^	0.84^†^	0.56^†^	0.72*	0.44	0.65	0.46	0.67	0.95	1.29	0.55^†^	0.72*
Eastern Europe	0.78^†^	0.90^†^	0.59^†^	0.65^†^	0.88	1.04	0.74	0.89	0.80^†^	0.9	0.60^†^	0.63^†^
Latin America	0.78^†^	0.92^†^	0.73^†^	0.86*	1.2	1.53*	0.87	1.02	0.71^†^	0.82*	0.42^†^	0.53^†^
Western Europe & North America	0.61^†^	0.78^†^	0.61^†^	0.73*	0.68	0.9	0.8	1.05	0.69*	0.86	0.52^†^	0.83*
Adults												
Nationals (ref)	1.00											
Immigrants	0.88^†^	0.95^†^	0.71^†^	0.74^†^	0.78^†^	0.83^†^	0.91^†^	0.94*	1.07^†^	1.16^†^	0.53^†^	0.54^†^
Africa	1.04^†^	1.10^†^	0.71^†^	0.70^†^	0.74^†^	0.76^†^	0.97	0.98	1.06*	1.11^†^	0.54^†^	0.56^†^
Asia	0.67^†^	0.84^†^	0.64^†^	0.74^†^	0.71*	0.81	0.92	1.02	1.02	1.24^†^	0.41^†^	0.47^†^
Eastern Europe	0.80^†^	0.90^†^	0.72^†^	0.76^†^	0.76^†^	0.83^†^	0.87*	0.93*	1.03*	1.14^†^	0.53^†^	0.55^†^
Latin America	0.91^†^	0.93^†^	0.90^†^	0.92^†^	0.86^†^	0.89^†^	0.94	0.96	1.25^†^	1.32^†^	0.49^†^	0.50^†^
Western Europe & North America	0.68^†^	0.79^†^	0.64^†^	0.66^†^	0.74^†^	0.81*	0.78*	0.82*	0.65^†^	0.73^†^	0.73^†^	0.71^†^

^a^Adjusted by age and sex ^b^Adjusted by age, sex and morbidity burden

^**¶**^In those cases where the Vuong test was statistically non-significant showing large negative values, standard negative binomial models were employed, *p < 0.05, ^†^ *p* < 0.001

Table 3 shows the IRR for use of emergency care and hospital care (unplanned admissions) among PC users and non-users. While immigrants who attended PC services showed significantly higher visit and admission rates, those not using PC services presented a lower rate of emergency room use and fewer unplanned hospital admissions. The only exception was Asian non-PC users, who showed a 50 % increase in the probability of visiting the emergency room compared to nationals.

**Table 3:** Use of Emergency Care and Hospital Care (unplanned admissions) among Primary Care users and non-users (incidence rate ratios, IRR). Results of standard or zero-inflated negative binomial regression models^¶^ across immigrant groups

	Use of Emergency Care				Use of Hospital Care			
	PC users		PC non-users		PC users		PC non-users	
	Mean No. of visits/10 ind.	IRR	Mean No. of visits/10 ind.	IRR	Mean No. of unplanned admissions/100 ind.	IRR	Mean No. of unplanned admissions/100 ind.	IRR
Nationals	3.13	(ref)	1.10	(ref)	4.60	(ref)	2.70	(ref)
Immigrants	3.28	1.20^†^	0.58	0.91^†^	4.02	1.15^†^	0.70	0.61^†^
Africa	3.72	1.08^†^	0.63	0.88*	4.10	1.25^†^	0.70	0.58^†^
Asia	3.44	1.36^†^	0.58	1.52^†^	3.73	1.47^†^	0.78	0.59
Eastern Europe	3.07	1.22^†^	0.64	0.88^†^	4.35	1.13^†^	0.74	0.57^†^
Latin America	3.32	1.32^†^	0.79	1.04	3.77	1.05	0.72	0.82
Western Europe & North America	2.76	0.94*	0.29	0.37^†^	4.14	1.12	0.61	0.26^†^

*PC* Primary Care; *Ind*. Individuals

All means are standardised by age and sex. Models adjusted by age, sex, and morbidity burden

^**¶**^In those cases where the Vuong test was statistically non-significant showing large negative values, standard negative binomial models were employed, *p < 0.05, ^†^ *p* < 0.001

**Fig. 1:**
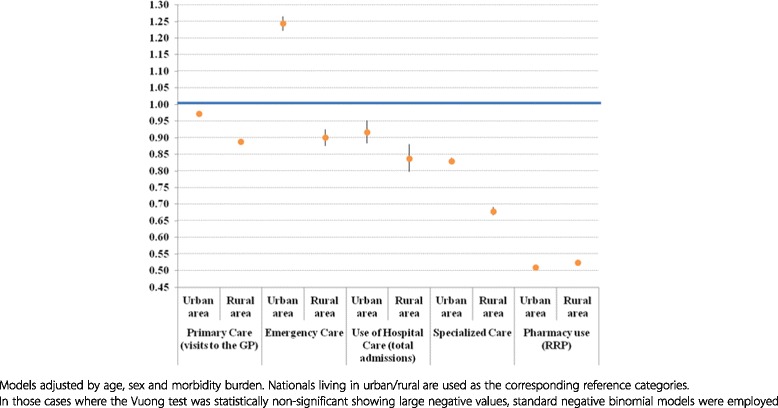
Use of healthcare services by immigrants living in urban vs rural areas (incidence rate ratios, IRR, and 95 % confidence intervals). Results of standard or zero-inflated negative binomial regression models^¶^

Finally, Fig. 1 depicts the urban/rural differences in the use of healthcare services for immigrants compared with nationals. Immigrants living in urban areas showed lower use rates than nationals for all levels of care except for emergency care. Furthermore, immigrants living in rural areas showed significantly lower use rates than those from urban areas, except in the case of hospital care, for which no significant differences were observed.

## Discussion

Our study shows that immigrants’ global use of health services, including prescription drug use, is significantly lower than that of Spanish nationals, even after adjusting by age, sex and morbidity burden, except for urban adult immigrants, who availed of emergency room services more frequently. Immigrants who did not use primary care services also showed lower rates of emergency service use and unplanned hospital admissions. Furthermore, we detected lower rates of health service use for immigrants living in rural versus urban areas. However, differences in the use of healthcare services were observed across immigrant groups depending on their area of origin.

### Strengths and Limitations

The strengths of this study include the region-wide coverage, the inclusion of all registered immigrants and the lack of selection bias, as well as the grouping of immigrants according to nationality, which recognises the heterogeneity of the immigrant population. Furthermore, by simultaneously analysing the use of primary care, specialised care, hospital, emergency room, and prescription drugs, this study constitutes a global assessment of the Spanish health system. This global approach provides a comprehensive overview of the main health resources involved, and allows us to determine whether underuse of one resource can provoke overuse of others. By measuring individual-level morbidity burden using an internationally validated tool and data from electronic medical records, we ensure a broad and reliable assessment of the health needs of the immigrant population [17, 25, 31–33]. Finally, the use of administrative data allowed us to study the effects of important socio-demographic characteristics such as length of stay, nationality, and rural/urban living.

Some limitations to the approach used should be noted. Our study did not consider socio-economic variables such as income, education level or legal status, which could have helped identify some of the complex factors that condition the use of healthcare services [19, 34–36]. This important personal information is not stored in Spanish healthcare databases and could not be obtained in any other way while preserving anonymity. The quality and completeness of the diagnostic data derived from PC registries and used to calculate patients’ morbidity burden is also open to question. However, these databases have been validated for use in population-level comparative studies [37] such as the present one. Moreover, comparison of our results with those obtained when morbidity burden categories were constructed using both PC and hospital diagnostic data showed few differences (data not shown).

### Main discussion points

Our study reveals a lower rate of health service use by immigrants as compared with Spanish nationals. These differences were only partly diminished after adjustment for morbidity burden, suggesting the existence of other non-clinical underlying factors.

The so-called “healthy migration effect” posits that the health of immigrants just after migration is substantially better than that of individuals in the host country [38–41]. This mainly applies to economic immigrants, which represent the majority of the immigrant population in Spain. However, after adjusting for morbidity burden, as repeatedly suggested in the literature [6, 16, 20, 42], our results indicate that for a given morbidity burden, immigrants’ use of health services is generally lower than expected. In agreement with a recent Norwegian study [16], we conclude that additional factors that influence health services utilisation rates should be investigated in future studies. Some studies have cited a lack of familiarity with rights, gaps in health literacy, direct and indirect discrimination, cultural barriers, a highly medicalized native culture, poor health system knowledge, and socio-economic inequality [2, 19, 22, 34, 38, 41, 43, 44] as factors that may account for the gap between health status and healthcare use among immigrants.

The lower PC use rates described here do not appear to be associated with higher hospitalisation rates, although they were linked with increased use of emergency room services by adults. However, this higher rate of emergency room use was only observed for immigrants living in urban areas, which may point to problems accessing urban PC services or cultural differences in the use of healthcare resources. Detailed analyses of the utilisation rates of immigrant non-PC users did not indicate higher emergency room use or a higher rate of unplanned hospital admissions. Potential explanations for these findings include better health among immigrants, barriers to the global use of healthcare services, and cultural differences in terms of the healthcare sought for a given condition [41]. Similarly, the lower use rates of specialised care and prescription drugs in immigrant populations, regardless of age, nationality, length of stay, or urban/rural setting, may be due to inequities in healthcare provision or a lower tendency to seek medical solutions for health problems.

The immigrant population is heterogeneous, as is their use of different healthcare services. In line with previous reports [7, 16, 17], the lowest use rates for all healthcare services were observed for patients from Western Europe and North America, while African, Asian and Latin American immigrants used some services significantly more often than Spanish nationals. Barriers to the use of services as well as differences in culture and health status may explain differences observed across groups. Another possible explanation is varying use of private healthcare services across groups of immigrants and Spanish nationals. Unfortunately this data was not available for analysis.

Among adult immigrants, length of stay in the host country was associated with higher rates of healthcare service use. McDonald and coworkers reported a higher frequency of use with increasing length of stay before health decline [38], suggesting an improved understanding of the health system or acculturation in terms of seeking healthcare with increasing time spent in the host country [41]. The reverse trend was observed for children. This cannot be easily explained and further research will be necessary to unravel the complex factors that underlie these patterns.

In Spain, family doctors visit even the smallest villages in order to ensure geographical accessibility [26]. As such, we did not expect to detect differences in PC use. However, significant rural/urban differences were observed for all healthcare services except for hospital admissions. Again, further studies will be required to explain these findings, preferably using qualitative methods.

## Conclusion

In a universal coverage health system accessible to all immigrants, global use of the healthcare system is lower in the immigrant population. Although the observed differences may be partially explained by the healthy migration effect, other factors should be promptly investigated to identify potential inequality factors. In particular, living in a rural area appears to be associated with a lower use of healthcare services by immigrants. The fact that healthcare use among adult immigrants increases with the length of stay in Spain while the opposite is observed in children warrants immediate attention to ensure equitable access to healthcare services.

## Abbreviations

ACG, Adjusted Clinical Group; ICD-9-CM, Ninth Revision of the International Classification of Diseases; ICPC-1, International Classification of Primary Care, Version 1; Ind, individuals; IRR, incidence rate ratios; PC, primary care; Ref, reference; RUB, resource utilisation bands; SD, standard deviation; WHO, World Health Organisation.

## Additional file

Additional file 1: Distribution of nationalities across immigrant groups. (DOCX 22 kb)
