# Supplementary material for: Global healthcare use by immigrants in Spain according to morbidity burden, area of origin, and length of stay
Source: BMC Public Health. 2016 May 27;16:450. doi: 10.1186/s12889-016-3127-5 (PMC4882823; doi:10.1186/s12889-016-3127-5)
Supplement: Additional file 1: — Distribution of nationalities across immigrant groups. (DOCX 22 kb) [file 12889_2016_3127_MOESM1_ESM.docx]

**Additional file 1- Distribution of nationalities across immigrant groups.**

|  | **N** | **%** |
| --- | --- | --- |
| **Latin America** | **41,060** |  |
| Ecuador | 13,073 | 31.84% |
| Colombia | 8,697 | 21.18% |
| Nicaragua | 2,802 | 6.82% |
| Peru | 2,756 | 6.71% |
| Dominican Republic | 2,658 | 6.47% |
| Argentina | 2,198 | 5.35% |
| Brazil | 1,991 | 4.85% |
| Cuba | 1,440 | 3.51% |
| Bolivia | 1,180 | 2.87% |
| Chile | 877 | 2.14% |
| Venezuela | 863 | 2.10% |
| Honduras | 678 | 1.65% |
| Uruguay | 604 | 1.47% |
| Mexico | 411 | 1.00% |
| Paraguay | 329 | 0.80% |
| El Salvador | 220 | 0.54% |
| Guatemala | 90 | 0.22% |
| Dominica | 64 | 0.16% |
| Panama | 56 | 0.14% |
| Costa Rica | 34 | 0.08% |
| Puerto Rico | 22 | 0.05% |
| Other countries | 17 | 0.04% |
| **Eastern Europe** | **56,011** |  |
| Romania | 44,375 | 79.23% |
| Bulgaria | 4,251 | 7.59% |
| Poland | 3,247 | 5.80% |
| Ukraine | 2,083 | 3.72% |
| Russia | 711 | 1.27% |
| Moldova | 364 | 0.65% |
| Lithuania | 334 | 0.60% |
| Slovakia | 163 | 0.29% |
| Hungary | 100 | 0.18% |
| Belarus | 84 | 0.15% |
| Albania | 68 | 0.12% |
| Georgia | 59 | 0.11% |
| Latvia | 42 | 0.07% |
| Bosnia and Herzegovina | 37 | 0.07% |
| Yugoslavia | 25 | 0.04% |
| Croatia | 23 | 0.04% |
| Slovenia | 20 | 0.04% |
| Serbia | 18 | 0.03% |
| Other countries | 7 | 0.01% |
| **Africa** | **37,603** |  |
| Morocco | 18,719 | 49.78% |
| Algeria | 5,200 | 13.83% |
| Senegal | 3,223 | 8.57% |
| Gambia | 2,281 | 6.07% |
| Ghana | 1,799 | 4.78% |
| Mali | 1,486 | 3.95% |
| Equatorial Guinea | 977 | 2.60% |
| Nigeria | 836 | 2.22% |
| Guinea | 732 | 1.95% |
| Mauritania | 466 | 1.24% |
| Cape Verde | 423 | 1.12% |
| Cameroon | 217 | 0.58% |
| Egypt | 208 | 0.55% |
| Guinea-Bissau | 155 | 0.41% |
| Burkina Faso | 115 | 0.31% |
| Ivory Coast | 112 | 0.30% |
| Republic of the Congo | 100 | 0.27% |
| Tunisia | 85 | 0.23% |
| Angola | 81 | 0.22% |
| Congo, Democratic Republic of | 44 | 0.12% |
| Sierra Leone | 47 | 0.12% |
| Niger | 40 | 0.11% |
| Western Sahara | 39 | 0.10% |
| Kenya | 31 | 0.08% |
| Liberia | 31 | 0.08% |
| Togo | 30 | 0.08% |
| Benin | 15 | 0.04% |
| Gabon | 14 | 0.04% |
| Mozambique | 15 | 0.04% |
| Central African Republic | 10 | 0.03% |
| Sudan | 13 | 0.03% |
| Other countries | 59 | 0.16% |
| **Asia** | **5,723** |  |
| China | 4,114 | 71.91% |
| Pakistan | 912 | 15.94% |
| Armenia | 135 | 2.36% |
| India | 100 | 1.75% |
| Syria | 58 | 1.01% |
| Turkey | 52 | 0.91% |
| Japan | 45 | 0.79% |
| Jordan | 37 | 0.65% |
| Philippines | 32 | 0.56% |
| Lebanon | 24 | 0.42% |
| Thailand | 22 | 0.38% |
| Uzbekistan | 21 | 0.37% |
| Iran | 19 | 0.33% |
| Bangladesh | 18 | 0.31% |
| Kazakhstan | 18 | 0.31% |
| Korea, North | 13 | 0.23% |
| Korea, South | 12 | 0.21% |
| Other countries | 91 | 1.59% |
| **Western Europe & North America** | **8,752** |  |
| Portugal | 3,753 | 42.88% |
| France | 1,309 | 14.96% |
| Italy | 1,257 | 14.36% |
| United Kingdom | 590 | 6.74% |
| Germany | 567 | 6.48% |
| Netherlands | 308 | 3.52% |
| United States | 287 | 3.28% |
| Belgium | 151 | 1.73% |
| Czech Republic | 129 | 1.47% |
| Switzerland | 73 | 0.83% |
| Ireland | 68 | 0.78% |
| Sweden | 45 | 0.51% |
| Greece | 39 | 0.45% |
| Austria | 37 | 0.42% |
| Denmark | 32 | 0.37% |
| Canada | 28 | 0.32% |
| Finland | 17 | 0.19% |
| Norway | 17 | 0.19% |
| Australia | 14 | 0.16% |
| Andorra | 11 | 0.13% |
| Other countries | 20 | 0.23% |
